# Interrogating the Role of the Two Distinct Fructose-Bisphosphate Aldolases of *Bacillus methanolicus* by Site-Directed Mutagenesis of Key Amino Acids and Gene Repression by CRISPR Interference

**DOI:** 10.3389/fmicb.2021.669220

**Published:** 2021-04-30

**Authors:** Kerstin Schultenkämper, Desirée D. Gütle, Marina Gil López, Laura B. Keller, Lin Zhang, Oliver Einsle, Jean-Pierre Jacquot, Volker F. Wendisch

**Affiliations:** ^1^Genetics of Prokaryotes, Faculty of Biology & CeBiTec, Bielefeld University, Bielefeld, Germany; ^2^INRAE, IAM, Université de Lorraine, Nancy, France; ^3^Institute for Biochemistry, Albert-Ludwigs-University Freiburg, Freiburg, Germany

**Keywords:** transketolase, methylotrophy, glycolysis, gluconeogenesis, CRISPR interference, fructose-1,6-bisphosphate aldolase, sedoheptulose 1,7-bisphosphate aldolase

## Abstract

The Gram-positive *Bacillus methanolicus* shows plasmid-dependent methylotrophy. This facultative ribulose monophosphate (RuMP) cycle methylotroph possesses two fructose bisphosphate aldolases (FBA) with distinct kinetic properties. The chromosomally encoded FBA^C^ is the major glycolytic aldolase. The gene for the major gluconeogenic aldolase FBA^P^ is found on the natural plasmid pBM19 and is induced during methylotrophic growth. The crystal structures of both enzymes were solved at 2.2 Å and 2.0 Å, respectively, and they suggested amino acid residue 51 to be crucial for binding fructose-1,6-bisphosphate (FBP) as substrate and amino acid residue 140 for active site zinc atom coordination. As FBA^C^ and FBA^P^ differed at these positions, site-directed mutagenesis (SDM) was performed to exchange one or both amino acid residues of the respective proteins. The aldol cleavage reaction was negatively affected by the amino acid exchanges that led to a complete loss of glycolytic activity of FBA^P^. However, both FBA^C^ and FBA^P^ maintained gluconeogenic aldol condensation activity, and the amino acid exchanges improved the catalytic efficiency of the major glycolytic aldolase FBA^C^ in gluconeogenic direction at least 3-fold. These results confirmed the importance of the structural differences between FBA^C^ and FBA^P^ concerning their distinct enzymatic properties. In order to investigate the physiological roles of both aldolases, the expression of their genes was repressed individually by CRISPR interference (CRISPRi). The *fba*^C^ RNA levels were reduced by CRISPRi, but concomitantly the *fba*^P^ RNA levels were increased. Vice versa, a similar compensatory increase of the *fba*^C^ RNA levels was observed when *fba*^P^ was repressed by CRISPRi. In addition, targeting *fba*^P^ decreased *tkt*^P^ RNA levels since both genes are cotranscribed in a bicistronic operon. However, reduced *tkt*^P^ RNA levels were not compensated for by increased RNA levels of the chromosomal transketolase gene *tkt*^C^.

## Introduction

*Bacillus methanolicus* MGA3 is a Gram-positive, thermophilic, methylotrophic bacterium originally isolated from freshwater marsh soil (Schendel et al., 1990; Arfman et al., 1992). Methylotrophs such as *B. methanolicus* utilize reduced one-carbon compounds as their sole sources of carbon and energy (Anthony, 1991; Chistoserdova et al., 2009; Chistoserdova, 2011). MGA3 can utilize methanol *via* the ribulose monophosphate (RuMP) cycle (Anthony, 1991; Arfman et al., 1992), a trait that makes it a promising candidate for biotechnological applications. *Bacillus methanolicus* is a methylotroph with a functionally active TCA cycle and glyoxylate shunt (Heggeset et al., 2012; Müller et al., 2015a; Delépine et al., 2020), although this is unusual since some methylotrophs, including some that use the RuMP cycle, do not require a complete TCA to cover their energy needs (Chistoserdova et al., 2009). ^13^C-labeling experiments revealed that the TCA cycle flux was lower during growth with methanol as substrate than with mannitol or arabitol (Delépine et al., 2020).

As a facultative methylotroph, *B. methanolicus* MGA3 can grow on a limited substrate spectrum besides methanol: metabolic pathways for the utilization of glucose and mannitol have been described (Heggeset et al., 2012), as well as the recent characterization of its fourth carbon and energy source arabitol (López et al., 2019). The increasing interest lays, however, on the production of fuels and chemicals from methanol, with its attractiveness laying on its low cost, abundant availability, and the reduced risks of microbial contamination during industrial fermentations due to the toxicity of the derivative formaldehyde (Dijkhuizen et al., 1985; Irla et al., 2015; Müller et al., 2015a). Additionally, methanol is not in competition with conventional feedstocks used in biotechnological processes. In order to improve our understanding of *B. methanolicus* metabolism, and specifically its methylotrophy, full sequencing of the genome (Heggeset et al., 2012; Irla et al., 2014) together with a series of studies at the transcriptomics (Irla et al., 2015; López et al., 2019), proteomics (Müller et al., 2014), and metabolomics (Müller et al., 2015b; Carnicer et al., 2016; Delépine et al., 2020) levels have already been carried out. Furthermore, to enable genetic manipulation for industrial purposes, tools for gene expression of *B. methanolicus* MGA3 have been developed: rolling circle- and theta-replicating plasmids for controlled gene overexpression (Irla et al., 2016). These advances have contributed to the application of *B. methanolicus* MGA3 for methanol-based production of l-glutamate and l-lysine (Brautaset et al., 2007), *gamma*-aminobutyric acid (GABA), the five-carbon diamine cadaverine (Naerdal et al., 2015; Irla et al., 2016), and the platform chemical (*R*)-acetoin (Drejer et al., 2020). The most recent addition to the restricted genetic toolbox of *B. methanolicus* has been the application of CRISPR interference (CRISPRi) (Schultenkämper et al., 2019), a relevant tool that opens the possibility for gene knock-down studies in this organism for the first time.

CRISPR interference is a genetic perturbation technique that allows for sequence-specific repression of gene expression in bacteria, archaea and eukaryotes (Qi et al., 2013). The tool has a huge impact on strain development and physiological screening of target genes (Schultenkämper et al., 2020). The CRISPRi system only requires the co-expression of a catalytically deactivated Cas9 protein (dCas9), which has two substitutions in the nuclease domains that render it inactive, and a customizable single guide RNA (sgRNA). The dCas9-sgRNA complex binds to DNA elements complementary to the sgRNA and causes a steric block that halts transcript elongation by RNA polymerase (RNAP), resulting in the repression of the target gene (Larson et al., 2013). If the target DNA sequence belongs to the protein-coding region, the dCas9–sgRNA–DNA complex blocks the movement of the RNAP and subsequent transcription elongation. Furthermore, the CRISPRi-dCas9 technology has already been developed and established in the model organisms *Escherichia coli* (Elhadi et al., 2016; Cress et al., 2017; Wu et al., 2017; Fontana et al., 2018; Sander et al., 2019), *Corynebacterium glutamicum* (Cleto et al., 2016; Zhang et al., 2016; Gauttam et al., 2019), *Bacillus subtilis* (Westbrook et al., 2016, 2018; Wu et al., 2018a; Wang et al., 2019), *Paenibacillus sonchi* genomovar Riograndensis SBR5 (Brito et al., 2020), and recently in *B. methanolicus* MGA3 (Schultenkämper et al., 2019).

The genome of *B. methanolicus* MGA3 is composed of a circular chromosome and the two naturally occurring plasmids pBM19 and pBM69 (Heggeset et al., 2012; Irla et al., 2014). The genes present in the pBM19 plasmid are required for its methylotrophy: sequence analysis of this plasmid showed the presence of one copy of the *mdh* gene, encoding a methanol dehydrogenase, which was shown to be crucial for methanol consumption in this bacterium (Brautaset et al., 2004). In addition, five additional genes [*pfk*, encoding phosphofructokinase; *rpe*, encoding ribulose-5-phosphate 3-epimerase; *tkt*, encoding transketolase; *glpX*, encoding fructose-1,6-bisphosphatase; and *fba*, encoding fructose-1,6-bisphosphate (FBP) aldolase] with deduced roles in methanol assimilation *via* the RuMP assimilation pathway are present in pBM19 (Brautaset et al., 2004). FBP aldolase (FBA) is an enzyme that catalyzes the reversible reaction of the aldol FBP cleavage into the triose phosphates dihydroxyacetone phosphate (DHAP) and glyceraldehyde 3-phosphate (GAP), thus representing an important enzyme of the glycolysis and gluconeogenesis pathways.

**Figure 1:**
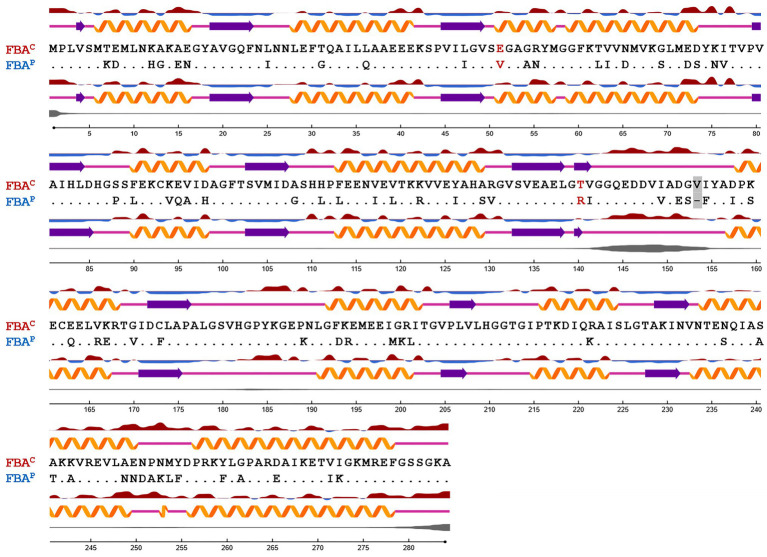
Sequence alignment based on the secondary structure of FBA^P^ and FBA^C^ from *B. methanolicus*. Protein secondary structure and surface accessibility predictions were performed with NetSurfP (Petersen et al., 2009; Klausen et al., 2019). The secondary structure is depicted with orange *α*-helices, purple *β*-strands, or coils with pink lines, respectively. Protein surface accessibility is shown by red (exposed) or blue (buried) waves, thresholder at 25%. Dots are representing identical amino acids. Amino acid residues exchanged by SDM are highlighted in red. A gray box depicts a gap in the amino acid sequence of FBA^P^ in comparison to FBA^C^.

**Figure 2:**
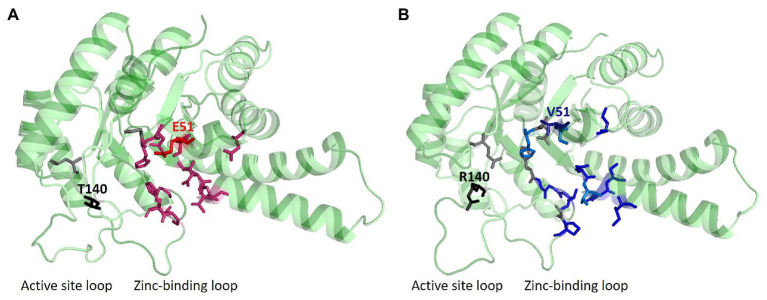
Ligand and structure prediction of FBA^C^ and FBA^P^. **(A)** 3D model of the FBA^C^ showing the protein structure, which was depicted with PyMOL (DeLano, 2002), and predicted ligands *via* COACH-D (Wu et al., 2018b) analysis. **(B)** 3D protein model of FBA^P^. In gray, residues that are important for the zinc-binding are shown; while in red residues that are necessary for binding the substrate FBP are plotted. Blue residues are representing important sites for GAP or DHAP interaction. Sites for SDM are marked in the 3D structures of the protein models (E51V, T140R). The 3D structure of the protein models indicates typical triosephosphate isomerase (TIM) barrels.

In the genome of *B. methanolicus* two *fba* genes encode FBA: *fba*^C^, which is located on the chromosome, and *fba*^P^, located on the natural plasmid pBM19 (Stolzenberger et al., 2013b). Aldolases can be divided into two classes depending on their mechanism of catalysis: class I aldolases, which form a Schiff base as an intermediate, and class II aldolases, which depend on divalent metal ions (Penhoet et al., 1966; Tittmann, 2014). *Bacillus methanolicus* FBAs belong to the latter group, and among the divalent metal ions that affect their activities it was found that manganese and cobalt acted as activators, while copper and EDTA completely inhibited their activities (Stolzenberger et al., 2013b). Based on the amino acid sequence, class II aldolases can be further divided into type A and type B (Plaumann et al., 1997; Shams et al., 2014). Thereby, type A enzymes are dimeric in contrast with the type B enzymes, which can be dimeric, tetrameric, or octameric (Sauvé and Sygusch, 2001; Nakahara et al., 2003). *Bacillus methanolicus* FBAs show a tetrameric subunit structure, thus belonging to the latter type B (Nakahara et al., 2003; Izard and Sygusch, 2004; Galkin et al., 2007). Usually the homotetramer structure of aldolases consists of a triosephosphate isomerase (TIM) beta/alpha-beta fold and the fold designation is based upon eight alpha helices and eight parallel beta-strands [(*α*/*β*)_8_] in a closed barrel of each monomeric subunit (Dalby et al., 1999; Wierenga, 2001; Wood, 2008). This typical structure is also called TIM barrel (Figures 1, 2) and is named after the TIM, a highly conserved metabolic enzyme (Banner et al., 1975). Mobility of the active site zinc is necessary to orient the catalytic aspartyl side chain and to polarize the substrate for proton transfer from the substrate FBP (Galkin et al., 2007, 2009). Additionally, kinetic parameters allowed to distinguish the *B. methanolicus* FBA^C^ as the major glycolytic aldolase and FBA^P^ as the major gluconeogenic one (Stolzenberger et al., 2013b).

In this study, crystal structures of both enzymes were obtained, at resolutions of 2.2 Å and 2.0 Å, respectively, which allowed identifying differences at crucial residues: glutamic acid (E) at position 51 and threonine (T) at position 140 were identified in FBA^C^ while, on the other hand, valine (V) at position 51 and arginine (R) at position 140 were identified in FBA^P^ (Figures 1, 2). The glutamic acid residue at position 51 (E51) is located at the FBP-binding site of FBA^C^, the FBP-cleaving major glycolytic FBA. Thus, exchanging the amino acid residues at position 51 from glutamic acid to valine (E51V) should affect the binding affinity of FBP (*K*_M_ value) as well as the glycolytic specific activity (*V*_max_). Position 140 coordinates zinc-binding, and when no substrate is around (apoenzyme), conformational changes near the zinc atom transform the apoenzyme into a holoenzyme, a crucial step for catalysis. The cofactor zinc coordinated in such a manner is involved in DHAP catalysis (Jacques et al., 2018). It is known that DHAP enters first into the catalytic centrum. Class II aldolases catalyze the aldol reaction by making use of divalent zinc or cobalt metal ions that is suggested to polarize the DHAP (or FBP in glycolysis) carbonyl group and stabilize the carbanion intermediate during catalysis (Belasco and Knowles, 1983; de la Paz Santangelo et al., 2011; Jacques et al., 2018). Hence, changing the position 140 from T to R should affect the gluconeogenic *V*_max_ and the *K*_M_ of DHAP as well as the glycolytic *V*_max_ and *K*_M_ of FBP.

To test these hypotheses and to gain further knowledge about the catalytic mechanisms of the *B. methanolicus* FBAs, the crystal structures of both FBA^C^ and FBA^P^ were solved and site-directed mutagenesis (SDM) was performed in order to exchange identified residues suggested to be crucial to interchange activities of FBA^C^ and FBA^P^. Both native enzymes and generated SDMs were examined in two different enzyme assays to test glycolysis and gluconeogenesis reactions. Moreover, gene expression analyses of *fba*^C^ and *fba*^P^ were performed with the help of the recently developed CRISPR interference technique in *B. methanolicus* and quantitative reverse transcription-polymerase chain reaction (qRT-PCR) analysis.

## Materials and Methods

### Structure Determination and Analysis

Amino acid sequences from *B. methanolicus* FBA^C^ (EU83897, AIE61578, and WP_03346852) and FBA^P^ (EIJ77593, AIE61790, and WP_003349819) from the National Center of Biotechnology Information (NCBI) were used for a multiple amino acid sequence alignment with BLASTp (Altschul et al., 1990) and their secondary structures and protein surface accessibility were predicted using NetSurfP (Petersen et al., 2009; Klausen et al., 2019). PyMOL files of the distinct FBA^C^ and FBA^P^ were computationally generated with PHYRE2 (Kelley et al., 2015). Sequences in FASTA format of FBA^C^ and FBA^P^ from *B. methanolicus* were used as input and the output file was analyzed with COACH-D (Wu et al., 2018b) to gain insights concerning the important residues for ligand binding. Finally, PyMOL (the PyMOL Molecular Graphics System, Version 1.8 Schrödinger, LLC; DeLano, 2002) was used for the depiction of the protein structures and the crucial residues for binding the substrates or zinc as a cofactor.

To obtain the crystal structures from the native *B. methanolicus* FBAs, the proteins were first purified. Consequently, *E. coli* strain BL21(DE3) was used for protein production (Studier and Moffatt, 1986). After a 4 h induction with 100 μM isopropyl-*β*-d-1-thio-galactopyranoside (IPTG), cells were harvested by centrifugation (5,000 × *g*, 10 min). After sonication (2 × 1 min) on ice and centrifugation (30,000 × *g*, 20 min, 4°C), the His-tagged enzymes were purified by IMAC column (GE Healthcare) process. Protein samples were stored in Tris-EDTA buffer pH 8. Proteins were crystallized by sitting-drop vapor diffusion. Crystals for FBA^C^ were obtained at 20°C in a reservoir containing 0.1 M sodium acetate trihydrate pH 4.5, 2.0 M ammonium sulfate, and a ratio of protein and reservoir with 25 and 75%, respectively. FBA^P^ crystals grew at 8°C with 50% protein and 50% reservoir containing 20% PEG 4000, 0.2 M imidazole/malate buffer at pH 6. Crystals were flash cooled in liquid nitrogen for storage and data collection. X-ray diffraction data of FBA^C^ were collected in-house at 100 K on a Rigaku MicroMax 007HF rotating anode X-ray generator equipped with amar research mar345dtb image plate detector. Data of FBA^P^ were collected on beamline X06DA with PILATUS 2 M-F detector (Dectris) at Swiss Light Source (Villigen, Switzerland). The FBA structures were solved by molecular replacement with MOLREP (Vagin and Teplyakov, 2010) using aldolase from *Bacillus anthracis* (PDB ID code 3Q94; Tan et al., 2011) as search model. Improvement of the initial model was carried out in cycles of refinement with REFMAC5 (Murshudov et al., 2011) and phenix.refine (Adams et al., 2010; Afonine et al., 2012), and manual rebuilding was done in COOT (Emsley and Cowtan, 2004). Data collection and refinement statistics are summarized in Supplementary Table S5.

### Strains, Media, and Culture Conditions

*E. coli* DH5α (Hanahan, 1983) was used as the standard cloning host. The strains used in this study are listed in Supplementary Table S1. *E. coli* strains were routinely cultivated at 37°C and 180 rpm in Lysogeny Broth (LB) medium or on LB agar plates supplemented with antibiotics (100 μg ml^−1^ ampicillin, 25 μg ml^−1^ chloramphenicol, and 50 μg ml^−1^ kanamycin) and 0.5 mM IPTG when necessary. *B. methanolicus* was used as expression host and cultivated at 50°C and 200 rpm in minimal MVcMY media as described in Brautaset et al. (2003) with 200 mM methanol and with 5 μg ml^−1^ chloramphenicol. For CRISPR interference, 12.5 mM mannitol was added as an inducer to the media to drive *dCas9* gene expression when required. Recombinant *B. methanolicus* strains were routinely plated on SOB agar plates with 5 μg ml^−1^ chloramphenicol. Main cultures of all *B. methanolicus* experiments were inoculated at a start optical density (OD_600_) of 0.1. Cultivations were performed in 500 ml baffled shake flasks with 50 ml media volume and in biological triplicates.

### Recombinant DNA Work

Molecular cloning was performed as described (Sambrook, 2001) using primer sequences listed in Supplementary Table S2. Total DNA isolation from *B. methanolicus* was performed following the indications of Eikmanns et al. (1994). Inserts were amplified by PCRs with ALLin™ HiFi DNA Polymerase (HighQu, Kraichtal, Germany) and purified using the NucleoSpin® Gel and PCR Clean-up kit (Macherey-Nagel, Düren, Germany). CRISPRi Plasmids were constructed using the isothermal DNA assembly method (Gibson et al., 2009; Gibson, 2011) with generated fragments by Annealing Oligo method (using the primer pairs for tfbaC and tfbaP, Supplementary Table S2), and the piCas vector cut with restriction enzymes (New England Biolabs, Ipswich, United Kingdom) as described in Schultenkämper et al. (2019). For plasmid isolation, the GeneJET Plasmid Miniprep Kit (Thermo Fisher Scientific, Waltham, United States) was used. Transformation of chemically competent *E. coli* cells was performed following the procedure described by Mandel and Higa (1970). Plasmids were transformed by polyethylene glycol-mediated procedure into *B. methanolicus* cells (Cue et al., 1997). FBA mutants were generated by SDM with PfuTurbo™ DNA Polymerase (Agilent, Böblingen, Germany). The generated PCR products were subsequently incubated with *Dpn*I to digest the parental non-mutated template. FBA^C; E51V^ and FBA^C; T140R^ were generated by backbone amplification of pET16b-*fba*^C^ using primer pairs SDM1_fwd and SDM1_rev and SDM2_fwd and SDM2_rev, respectively, while FBA^P; V51E^ and FBA^P; R140T^ were generated by backbone amplification of pET16b-*fba*^P^ using primer pairs SDM3_fwd and SDM3_rev and SDM4_fwd and SDM4_rev, respectively (Supplementary Table S3). The double mutants FBA^C; E51V, T140R^ and FBA^P; V51E, R140T^ were constructed based on the single mutants FBA^C; E51V^ and FBA^P; R140T^, respectively. The amino acid sequences are listed in Supplementary Table S4. A detailed description of each performed mutation, the respective plasmid templates and primer pairs used can be found in Supplementary Table S3. All cloned DNA fragments were verified by sequencing (Sequencing Core Facility, Bielefeld University).

### CRISPRi Targeting of *fba*^C^ and of *fba*^P^

Our recently developed piCas plasmid system (Schultenkämper et al., 2019) was used to target *fba*^C^ and *fba*^P^ in *B. methanolicus*. The piCas plasmid was linearized with *Ava*I and *Xba*I double restriction and with help of the CRISPy webtool (Blin et al., 2016) the most efficient target sequence (antisense strand) was chosen for *fba*^C^ and *fba*^P^ (Supplementary Table S2) as described previously (Schultenkämper et al., 2019). The plasmids piCas-*tfba*^C^ and piCas-*tfba*^P^ were transformed as described above into *B. methanolicus*, and cells were then cultivated in MVcMY media with 200 mM methanol and 12.5 mM mannitol for the induction of the dCas9 system when needed. When the cells reached an OD_600_ of 1.0, they were harvested and prepared based on the protocol described by Brautaset et al. (2004) and used for further enzymatic assays and RNA isolation.

### Protein Overproduction and Purification

Plasmids for the production of N-terminal decahistidine-tagged FBA proteins using *E. coli* BL21 (DE3) were previously constructed based on pET16b (Novagen, Madison, WI, United States; Stolzenberger et al., 2013a) and subsequently used in this work for SDM experiments. The already available pET28a-*fbp* plasmid was used for the production of the N-terminal His-tagged GlpX from *C. glutamicum* (Rittmann et al., 2003a), which was required as a coupling enzyme in several of the enzymatic assays performed. The plasmids used in this study are listed in Supplementary Table S1. Protein production and purification was performed following the procedure described by Lindner et al. (2007) except for cell lysis, which was performed by sonication (UP 200 S, Dr. Hielscher GmbH, Teltow, Germany) on ice at an amplitude of 55% and a duty cycle of 0.5 for 8 min. Supernatants were subsequently filtered using a 0.2 μm filter and purified by nickel affinity chromatography with nickel-activated nitrilotriacetic acid-agarose (Ni-NTA; Novagen, San Diego, CA, United States). GlpX and the distinct FBA variants eluted with 20 mM Tris, 300 mM NaCl, 5% (vol/vol) glycerol, and 50, 100, 200, or 400 mM imidazole were analyzed by 12% SDS-PAGE (Laemmli, 1970). Fractions showing the highest protein concentrations (i.e., eluted with 100 and 200 mM imidazole) were pooled and buffered in 50 mM Tris-HCl (pH 7.5) using Vivaspin® columns (10,000 MW, Sartorius, Göttingen, Germany). After purification, the His-tag was cleaved using the Factor Xa Cleavage Capture Kit (Novagen, San Diego, CA, United States) following the manufacturer’s recommendations (Supplementary Figure S1). Protein concentration was measured according to the Bradford method (Bradford, 1976) using bovine serum albumin (BSA) as a reference. The purified proteins were subsequently applied for enzymatic assays.

### Enzyme Activity Assays

Activity measurements of the different FBA variants in the glycolytic and gluconeogenic directions were performed following the indications of Stolzenberger et al. (2013b). Determination of the FBA activity in the direction of FBP cleavage was performed using an NADH-linked enzyme assay with the coupling enzyme *α*-glycerol 3-phosphate dehydrogenase (G3PDH) from rabbit muscle (Sigma-Aldrich, St. Louis, MO, United States) and FBP as substrate. The assay mixture contained 50 mM Tris-HCl buffer (pH 7.5), 0.25 mM NADH, 5 U G3PDH, and 0.05–20 mM FBP to initiate the reaction and varying concentrations of the FBA variants, which were pre-warmed for 4 min at 50°C, in a total volume of 1 ml. The oxidation rate of NADH was monitored at 340 nm and 50°C for 3 min using a Shimadzu UV-1202 spectrophotometer (Shimadzu, Duisburg, Germany). Determination of the FBA activity in the direction of FBP synthesis toward 6-phosphogluconolactone (6PGL) was performed by an NADPH-linked enzyme assay with the coupling enzymes phosphoglucoisomerase (PGI) from *Saccharomyces cerevisiae* (Sigma-Aldrich, St. Louis, MO, United States), glucose-6-phosphate dehydrogenase (G6PDH) from *Leuconostoc mesenteroides* (Sigma-Aldrich, St. Louis, MO, United States), purified GlpX (FBPase) from *C. glutamicum*, and DHAP and GAP as substrates. The assay mixture contained 50 mM Tris-HCl buffer (pH 7.5), 0.25 mM NADP^+^, 5 U PGI, 5 U G6PDH, 1.5 U GlpX, 50 mM KCl, 2 mM MnCl_2_, and 0.5–5 mM DHAP and GAP to initiate the reaction and varying concentrations of the FBA variants, which were again pre-warmed for 4 min at 50°C, in a total volume of 1 ml. In order to determine the kinetic parameters of GAP and DHAP, one of the substrates’ concentration was left constant while the other substrate concentration was varied (Michaelis et al., 2011). The reduction rate of NADP^+^ was monitored at 340 nm and 50°C for 3 min using a Shimadzu UV-1202 spectrophotometer (Shimadzu, Duisburg, Germany).

### Gene Expression Analysis Using qRT-PCR

Total RNA was isolated from *B. methanolicus* MGA3(piCas-*tfba*^C^) and MGA3(piCas-*tfba*^P^) cells growing exponentially (at OD_600_ = 1.0) in MVcMY medium containing 200 mM methanol and 12.5 mM mannitol to induce *dcas9* expression. The pellets were stored at −80°C. For the RNA isolation, the pellets were thawed on ice, and the samples were homogenized by resuspending the cells in 100 μl TE buffer (10 mM Tris-HCl, 1 mM EDTA; pH 8) containing 5 mg ml^−1^ lysozyme. After an incubation step at 37°C for 30 min, total RNA was extracted using NucleoSpin® RNA kit (Macherey-Nagel) according to the manufacturer’s instructions. Thereafter, RNA samples were treated with DNase digestion using RNase-free DNase Set and RNeasy MinElute kits (Qiagen, Hilden, Germany) to eliminate possible genomic DNA contamination. Furthermore, quality control was performed in order to determine the purity and integrity of isolated RNA. A series of PCRs were performed using Taq polymerase (New England Biolabs) amplifying regions of sizes between 1,000 and 2,000 bp. Additionally, RNA concentration was measured using a spectrophotometer (NanoDrop®, ND-1000). Fifty nanograms of each sample were used to perform cDNA synthesis. All qRT-PCRs were performed according to the manufacturer’s instructions using the SensiFAST™ SYBR® No-ROX One-Step Kit (Bioline, London, United Kingdom) and the CFX96 cycler system (Bio-Rad, Hercules, United States). The temperature profile was (1) 45°C for 10 min (reverse transcription); (2) 95°C for 2 min; (3) 40 cycles of 95°C for 5 s, 55°C for 10 s, and 72°C for 5 s; and (4) melt curve analysis with measures between 65 and 95°C. The ΔΔCt method was used in calculations (Higuchi et al., 1992; Pfaffl, 2001; Bustin et al., 2009) with the reference gene *parA* (Plasmid partition protein A) and empty vector as control. For each sample, three independent qRT-PCR experiments were performed.

## Results

### Structural Comparison of Native FBA^C^ and FBA^P^

The *B. methanolicus* class II type B enzymes FBA^P^ and FBA^C^ that share 75% identical amino acids differ considerably regarding their kinetic parameters (Stolzenberger et al., 2013b). To gain insight into the structure-function relationship of these enzymes, they were crystallized, and their structures were solved to 2.2 Å and 2.0 Å, respectively. Secondary structure elements are mapped onto the amino acid sequence in Figure 1. Approximately, 46% of the residues in the model are in helical conformations and 16% are in *β* strands. This can also be observed in the overall fold of the FBA protomer structure in Figure 2. The tetrameric FBA is composed of two pairs of dimers. The core of the structure consists of an eight-stranded parallel β-strand, twisted of strand–helix–strand winds, and building the typical barrel structure. The residues E51 and V51, in FBA^C^ and FBA^P^ respectively, were proposed to play an important role in recognition of the substrate FBP. The crystal structures show that this residue is located at the N-terminus of strand β3 of one subunit, directed into the middle of the TIM barrel with FBP bound at the active site of the partner subunit. It is assumed that the DHAP component will therefore be positioned at the other end of the active site near the catalytic zinc ion (Figure 2, gray and black residues; Cooper et al., 1996). The residues T140 and R140, of FBA^C^ and FBA^P^, respectively, are in the active center. Since the residues differ at positions 51 and 140 between FBA^P^ and FBA^C^ they may play a role in their different kinetic parameters. Therefore, SDM was performed, and three variants of each enzyme were generated and purified: FBA^C; E51V^, FBA^C; T140R^, FBA^C; E51V, T140R^, FBA^P; V51E^, FBA^P; R140T^, and FBA^P; V51E, R140T^ (Supplementary Table S3). His-tagged proteins were purified from recombinant *E. coli* and used for enzymatic characterization after His-tag cleavage.

### Influence of Amino Acid Residues 51 and 140 of FBA^P^ and FBA^C^ on Their Glycolytic Aldol Cleavage Activities

In order to test if changing the amino acid residues relevant for FBP and zinc ion binding negatively affect the glycolytic aldol cleavage activity of the major glycolytic aldolase FBA^C^, the mutants FBA^C; E51V^, FBA^C; T140R^, and FBA^C; E51V,T140R^ were analyzed. The amino acid exchange E51V did not increase the *K_M_* value for FBP (i.e., decrease in affinity), while the change T140R and changing both residues increased the *K_M_* value for FBP two- to three-fold (Table 1). The *k_cat_* was reduced about 2-fold when changing these residues individually, but the effect was not additive (Table 1). In comparison to the native aldolase FBA^C^, the double mutant FBA^C; E51V, T140R^ showed about 6 to 7-fold reduced catalytic efficiency for FBP cleavage. Compared to the major gluconeogenic aldolase FBA^P^, the double mutant showed a comparable *k_cat_*, but a 3-fold lower *K_M_* value for FBP. Thus, exchanging the two amino acid residues E15 and T140 relevant for FBP and zinc ion binding in FBA^C^ were almost sufficient to reduce its glycolytic aldol cleavage activity to that of the native major gluconeogenic aldolase FBA^P^.

**Table 1:** Kinetic parameters [fructose-1,6-bisphosphate (FBP) cleavage] of native FBAs and site-directed mutagenesis (SDMs) in glycolysis.

Sample	*V*_max_ (U mg^−1^)	*K*_M_ (FBP, mM)	*k*_cat_ (s^−1^)	Catalytic efficiency (s^−1^ mM^−1^)
FBA^C^	4.2	0.3	2.1	6.9
FBA^C; E51V^	2.8	0.2	1.4	7.2
FBA^C; T140R^	2.4	0.7	1.3	1.8
FBA^C; E51V,T140R^	2.2	1.0	1.1	1.1
FBA^P^	1.8	3.4	1.0	0.3
FBA^P; V51E^	0.5	5.8	0.3	0.1
FBA^P; R140T^	0.4	4.7	0.2	<0.1
FBA^P; V51E, R140T^	0.01	<0.01	<0.01	<0.01

In order to test if changing the amino acid residues relevant for FBP and zinc ion binding increases glycolytic aldolase activity of the major gluconeogenic aldolase FBA^P^, the mutants FBA^P; V51E^, FBA^P; R140T^, and FBA^P; V51E, R140T^ were analyzed. Surprisingly, these changes led to a complete loss of FBP aldol cleavage activity rather than increasing it (Table 1). It has to be noted that the proteins are known to be active since they showed activity in the reverse gluconeogenic direction (see below). Thus, exchanging these amino acids in the FBA^P^ backbone was not sufficient to increase its FBP aldol cleavage activity.

### Influence of Amino Acid Residues 51 and 140 of FBA^P^ and FBA^C^ on Their Gluconeogenic Aldol Condensation Activities

Coupled enzyme activity assays were performed with the purified native enzymes and their SDM variants to quantify aldol condensation of GAP and DHAP to FBP. The coupled activity assay to determine gluconeogenic FBP synthesis from GAP and DHAP required three helping enzymes. While PGI from *S. cerevisiae* and G6PDH from *L. mesenteroides* are available commercially, GlpX had to be purified from *C. glutamicum*. It showed a specific GlpX activity of 6.9 ± 0.6 U mg^−1^ at 50°C and was used to assay FBP synthesis from GAP and DHAP.

In order to test if the gluconeogenic aldol condensation activity of the major gluconeogenic aldolase FBA^P^ is negatively affected upon changing the amino acid residues relevant for FBP and zinc ion binding, the mutants FBA^P; V51E^, FBA^P; R140T^, and FBA^P; V51E, R140T^ were analyzed. The amino acid exchange in the FBP binding site (V51E) resulted in an increased *K_M_* value for GAP as substrate, while that for the substrate DHAP was in a much lower range (Table 2). The *k_cat_* values were reduced about 2-fold for both substrates as a consequence of the R140T exchange in the zinc ion binding site in the active center (Table 2). It has to be noted that the FBA^P^ variant with both amino acid exchanges (FBA^P; V51E, R140T^) was active in the gluconeogenic direction at a rate similar to the major glycolytic aldolase FBA^C^ (Table 2), while no significant activity in the glycolytic direction could be detected for this mutant (Table 1).

**Table 2:** Kinetic Parameters (FBP synthesis) of native FBAs and SDMs in gluconeogenesis.

GAP as substrate					DHAP as substrate			
Sample	*V*_max_ (U mg^−1^)	*K*_M_ (mM)	*k*_cat_ (s^−1^)	Catalytic efficiency (s^−1^ mM^−1^)	*V*_max_ (U mg^−1^)	*K*_M_ (mM)	*k*_cat_ (s^−1^)	Catalytic efficiency (s^−1^ mM^−1^)
FBA^C^	2.9	0.5	1.5	3.0	3.6	0.2	1.8	9.0
FBA^C; E51V^	10.1	0.2	5.2	25.8	21.8	3.3	11.1	3.4
FBA^C; T140R^	8.9	0.2	4.5	22.7	6.1	0.5	3.1	6.2
FBA^C; E51V, T140R^	9.0	0.2	4.6	23.0	5.6	0.1	2.9	28.6
FBA^P^	4.8	0.3	2.6	8.6	4.0	0.5	2.1	4.3
FBA^P; V51E^	2.9	1.0	1.6	1.6	4.1	0.8	2.2	2.7
FBA^P; R140T^	2.5	0.4	1.3	3.3	2.2	0.3	1.2	3.9
FBA^P; V51E, R140T^	2.1	0.3	1.1	3.7	2.7	0.3	1.4	4.8

In order to test if the gluconeogenic aldol condensation activity of the major glycolytic aldolase FBA^C^ can be increased by changing the amino acid residues relevant for FBP and zinc ion binding, the mutants FBA^C; E51V^, FBA^C; T140R^, and FBA^C; E51V,T140R^ were analyzed. The amino acid exchange in the FBP binding site (E51V) reduced the *K_M_* value for the substrate GAP about 2-fold but increased that for the substrate DHAP more than 10-fold (Table 2). This amino acid exchange also increased the *k_cat_* values 3- and 6-fold for GAP and DHAP, respectively (Table 2). The amino acid exchange T140R also increased the *k_cat_* values about 2- and 3-fold (Table 2).

The FBA^C^ double mutant FBA^C; E51V, T140R^ showed catalytic efficiencies of 28.6 and 23.0 s^−1^ mM^−1^ for GAP and DHAP, respectively. Thus, the combined introduction of the amino acid exchanges E51V and T140R into the major glycolytic aldolase FBA^C^ improved the catalytic efficiencies for the substrates GAP and DHAP about 7- and 3-fold, respectively. Notably, the catalytic efficiencies of FBA^C; E51V, T140R^ also exceeded those of FBA^P^, the major native gluconeogenic aldolase of MGA3.

### Repression of *fba*^C^ and *fba*^P^ by CRISPR Interference

**Figure 3:**
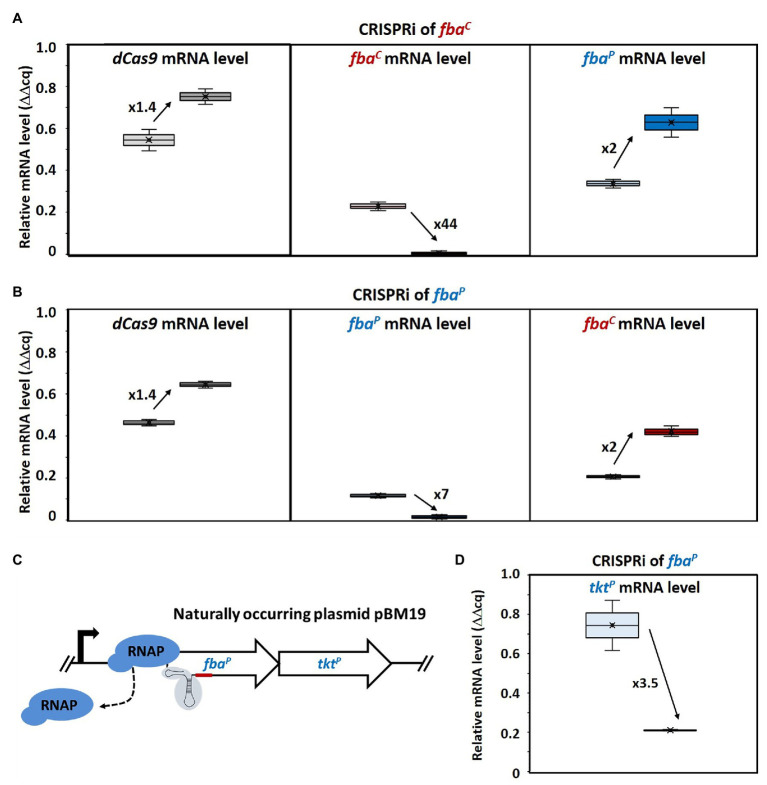
Changed gene expression levels due to dCas9 targeting *fba*^C^ and *fba*^P^ and polar effect on *tkt*^P^ by CRISPRi targeting of *fba*^P^. Relative mRNA levels of *dCas9* (left), *fba*^C^ (middle), and *fba*^P^ (right) in MGA3(piCas-*tfba*^C^) **(A)** and relative mRNA levels of *dCas9* (left), *fba*^P^ (middle), and *fba*^C^ (right) in MGA3(piCas-*tfba*^P^) **(B)** are shown by Box and Whisker plots as detected by qRT-PCR. Schematic representation of CRISPRi targeting *fba*^P^, which is cotranscribed with *tkt*^P^ as bicistronic operon on the natural plasmid pBM19 of *B. methanolicus* MGA3 **(C)**. CRISPR interference by blocking transcription elongation by RNA polymerase (RNAP; blue) is shown. qRT-PCR analysis of *tkt*^P^ mRNA levels upon CRISPRi targeting of *fba*^P^ in MGA3(piCas-*tfba*^P^) **(D)**. Black arrows with adjacent numbers indicate up- or downregulation without (lighter color, left) and with (darker color, right) CRISPRi induction.

In order to characterize the roles of FBA^C^ and FBA^P^ for *B. methanolicus* MGA3, expression of their genes was repressed individually by CRISPRi, and the effects on growth, on aldolase enzyme activity in crude extracts, and *fba*^C^ and *fba*^P^ RNA levels were determined. Cultivation of MGA3(piCas-*tfba*^C^) and MGA3(piCas-*tfba*^P^) revealed no growth deficit upon *dcas9* induction with mannitol (Supplementary Figure S2). A *dcas9* specific qRT-PCR analysis showed that *dcas9* expression was induced upon mannitol addition (Figures 3A,B).

Consequently, mRNA levels of the targeted *fba*^C^ gene in MGA3(piCas-*tfba*^C^) were 44-fold lower when mannitol was added as an inducer (Figure 3A). Surprisingly, increased mRNA levels (2-fold) of the plasmid-encoded gene *fba*^P^ were also observed in this strain. Vice versa, in strain MGA3(piCas-*tfba*^P^), the mRNA levels of the targeted *fba*^P^ were reduced 7-fold upon induction whereas the mRNA level of the chromosomal *fba*^C^ was 2-fold higher (Figure 3B). This indicated a compensatory effect at the mRNA level. We aimed then at determining how this compensatory effect at the mRNA level would affect enzyme activity levels. To this end, aldolase enzyme assays with crude extracts of MGA3(piCas-*tfba*^C^) and MGA3(piCas-*tfba*^P^) were performed in the FBP cleavage direction. It has to be noted that assaying crude extracts for fructose bisphosphate aldolase activity will yield the sum of both FBA^C^ and FBA^P^ activities. The aldolase enzyme activity detected in crude extracts did not change for strain MGA3(piCas-*tfba*^P^) under induced as compared to non-induced conditions, while the activity detected was higher upon induction for strain MGA3(piCas-*tfba*^C^) (Table 3). This confirmed a compensatory effect, and in the case of strain MGA3(piCas-*tfba*^C^), an overcompensation was observed. Since FBA^C^ and FBA^P^ differ in their affinity for FBP, the crude extracts were analyzed to derive an estimate of the *K_M_* value for FBP (Table 3). The *K_M_* value estimated using the crude extracts of strain MGA3(piCas-*tfba*^C^) increased by 20-fold upon induction. This may be explained if repression of *fba*^C^ reduced the levels of the low-*K_M_* aldolase FBA^C^ (*K_M_* value of 0.3 mM for FBP for the purified enzyme) and if compensatory induction of *fba*^P^ yielded higher levels of the high-*K_M_* aldolase FBA^P^ (*K_M_* value of 3.4 mM for FBP for the purified enzyme).

**Table 3:** FBA (FBP cleavage) activities in crude extracts of *Bacillus methanolicus* MGA3(piCas-*tfba*^C^) and MGA3(piCas-*tfba*^P^) without (−) and with (+) induction of CRISPR interference (CRISPRi).

Strain	Induction	*V*_max_ (U mg^−1^)	*K*_M_ (FBP, mM)
MGA3(piCas-*tfba*^C^)	−	0.1 ± 0.03	0.1 ± 0.01
MGA3(piCas-*tfba*^C^)	+	0.3 ± 0.04	2.0 ± 0.05
MGA3(piCas-*tfba*^P^)	−	0.1 ± 0.02	0.2 ± 0.02
MGA3(piCas-*tfba*^P^)	+	0.1 ± 0.03	0.2 ± 0.03

CRISPR interference of a gene in an operon may affect the expression of downstream genes. Since *fba*^P^ is encoded in a bicistronic operon on the naturally occurring plasmid pBM19 in *B. methanolicus* and cotranscribed with transketolase gene *tkt*^P^ (Figure 3C), *tkt*^P^ mRNA levels were determined in the MGA3(piCas-*tfba*^P^) strain targeting *fba*^P^. Indeed, a polar effect on *tkt*^P^ was observed when *fba*^P^ was targeted by CRISPRi since the *tkt*^P^ mRNA levels were reduced by almost 4-fold (Figure 3D).

**Figure 4:**
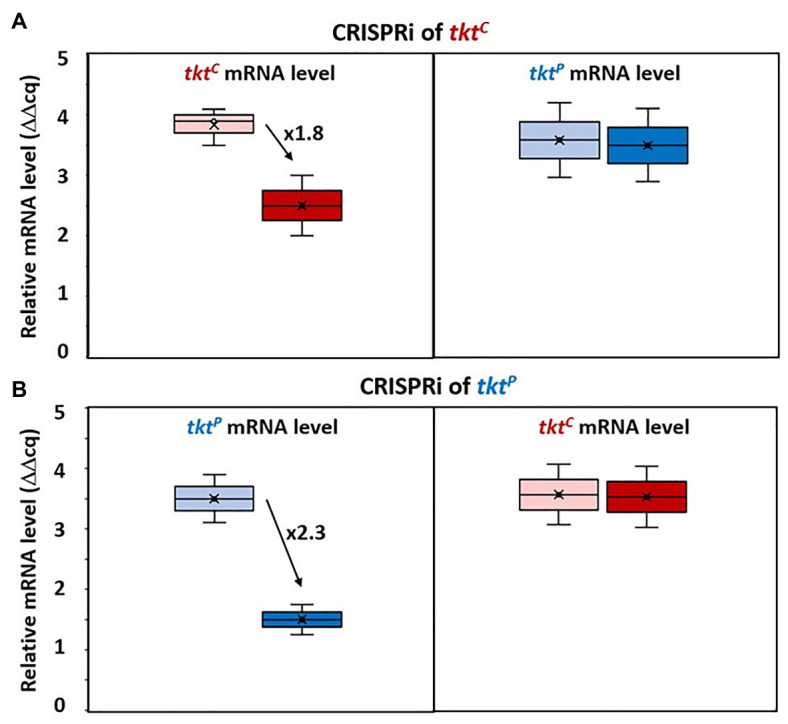
Changed gene expression levels due to dCas9 targeting *tkt*^C^ and *tkt*^P^. Relative mRNA levels of *tkt*^C^ (left) and *tkt*^P^ (right) in MGA3(piCas-*ttkt*^C^) **(A)** and relative mRNA level of *tkt*^P^ (left) and *tkt*^C^ (right) in MGA3(piCas-*ttkt*^P^) **(B)** are shown by Box and Whisker plots as detected by qRT-PCR. Black arrows with adjacent numbers indicate up- or downregulation without (lighter color, left) and with (darker color, right) CRISPRi induction.

*B. methanolicus* possesses a second transketolase gene encoded on its chromosome by the monocistronic gene *tkt*^C^. To examine if the expression of the transketolase genes may also be regulated by compensation as observed for the aldolase genes *fba*^C^ and *fba*^P^, *tkt*^P^ and *tkt*^C^ were subjected to CRISPRi targeting (Figures 4A,B). CRISPRi targeting *tkt*^C^ repressed *tkt*^C^ almost 2-fold, while qRT-PCR analysis revealed an unchanged mRNA level of *tkt*^P^ (Figure 4A). Similarly, CRISPRi targeting *tkt*^P^ did not affect *tkt*^C^ mRNA levels, but reduced *tkt*^P^ mRNA levels more than 2-fold (Figure 4B). Thus, a compensatory regulation at the mRNA level was not observed for the transketolase genes *tkt*^C^ and *tkt*^P^.

## Discussion

With the help of CRISPRi and SDMs, the characterization of FBA^C^ and FBA^P^ of *B. methanolicus* was significantly improved in this work. Using the SDM approach, we could show that exchange of the chosen amino acid residues in order to alternate FBA activity supported previous reports on the distinct roles displayed by FBA^C^ and FBA^P^. Furthermore, CRISPRi revealed a cooperative operation of the two FBAs, since higher gene expression of the untargeted *fba* could be observed when the other *fba* gene was repressed, and vice versa. In addition, we could show the effect of CRISPRi-derived repression of gene expression of a gene downstream of the target gene belonging to the same operon for the first time in *B. methanolicus*.

FBA is a key enzyme in glycolysis and gluconeogenesis in many organisms, and in *B. methanolicus* it plays a crucial role in the assimilation of methanol through the RuMP cycle. In the evolution of FBA enzymes, it is known that SBPases are of archaeal origin, whereas FBPases are descended from bacteria (Gütle et al., 2016, 2017). Class II FBAs catalyze the second reversible step of the glycolytic pathway, producing GAP and DHAP from the cleavage of the open-chain form of FBP (Marsh and Lebherz, 1992; Pegan et al., 2013). Little to no information has been reported on the interaction of class II or bacterial FBA aldolases with the substrate FBP, thus, there is a clear motivation to study this interaction in more detail. Recently, the X-ray structure of class II FBA from *Mycobacterium tuberculosis* (MtFBA) was determined with the aim to use it as a new pharmacological target against tuberculosis, since the class II MtFBA differs from the class I FBA in humans (Pegan et al., 2013). In addition, surface labeling and enzyme activity measurements revealed that MtFBA was exported to the cell surface of *M. tuberculosis* and produced under various axenic growth conditions, including oxygen deficiency (de la Paz Santangelo et al., 2011). However, the exact binding mechanism of class II FBAs to its substrate FBP is not yet fully understood. In this work, it could be shown through an SDM approach that we were able to alternate and significantly improve the catalytic efficiency of FBA^C^ in gluconeogenesis in the double mutant FBA^C; E51V,T140R^, even surpassing that of native FBA^P^ by at least 3-fold (Table 2). On the other hand, instead of the expected alternation from major gluconeogenic to major glycolytic aldolase (Supplementary Figure S3), a complete loss of glycolytic activity of FBA^P^ could be observed when performing both V51E and R140T amino acid exchanges (FBA^P;V51E, R140T^). Nevertheless, these results strongly indicate that the chosen amino acid residues are important for glycolytic activity and suggest that the mutant FBA^P; V51E, R140T^ is a strict gluconeogenic enzyme.

As previously reported (Galkin et al., 2007; Stolzenberger et al., 2013b), the presence of zinc decreased the FBA^C^ and FBA^P^ activity significantly. Therefore, the zinc-binding site was additionally chosen for mutagenesis purposes as it represented a strong candidate for the activity alternation. Single mutants FBA^C; T140R^ and FBA^P; R140T^ were constructed and investigated with regard to their enzyme activity. However, the obtained results indicate that the performed mutations at the zinc-binding site are not crucial to change the binding affinity of the substrates, since the observed *K_M_* values of mutants in the gluconeogenic direction did not differ from that of the native proteins, and only a slight decrease in the binding affinity of FBA^P; R140T^ for FBP in the glycolytic direction could be observed. Taken together, these results strongly indicate that the amino acid residue divergence at the FBP binding site is crucial for the reactivity change since *K_M_* values for FBA^P; V51E^ for the substrates FBP, GAP, and DHAP increased dramatically in comparison to the native protein (Table 1 and Table 2).

Moreover, the Hill coefficients determined (data not shown) indicate that the affinity of binding an FBP molecule is not dependent on whether other FBP molecules are already bound to FBA. This is based on the observation that Hill coefficient values are ≈ 1 for all FBA variants. Furthermore, the Lineweaver Burk Plots generated to determine the kinetic parameters in gluconeogenesis (Supplementary Figures S4, S5) for two different substrates indicate that the FBAs follow the rules of an equilibrium-ordered mechanism (Fromm, 1976), which describes sequential substrate binding to the enzyme. Once all substrates are bound, there is a central complex where the conversion of the substrates to the products takes place, which is subsequently released from the complex (Gates and Northrop, 1988). Since the crossing points to determine the *K_M_* and *V_max_* values for DHAP are in the second quadrant and since the crossing points for GAP as substrates are located close to the ordinate, it can be assumed that DHAP has to bind the FBA enzyme before the substrate GAP is able to bind, as reported by Segel (1975) and Fromm (1976). In addition, Rozovsky and McDermott (2007) used solid-state and solution-state nuclear magnetic resonance (NMR) to analyze the identity and steady-state populations of chemical entities bound to the TIM barrel. It can be assumed that the predominance of DHAP on the enzyme would support a mechanism where the initial proton abstraction in the reaction from DHAP to GAP is significantly slower than the subsequent chemical steps. This is also supported by the fact that most calculated *V_max_* values for DHAP as substrate were lower than those for GAP (Table 2).

Regarding the physiological role of FBA, it is reported that the FBA has a general “household function,” which is to maintain a rapid equilibrium between FBP, GAP, and DHAP. Further, this ensures the rapid equilibrium of triose phosphates produced by aldolase in glycolysis, which is interconnected to lipid metabolism, the GAP shuttle, and the pentose phosphate pathway (PPP) (Orosz et al., 2009; Malabanan et al., 2012). The major gluconeogenic aldolase FBA^P^ is expected to play an important role during growth of *B. methanolicus* with carbon sources requiring gluconeogenesis such as acetate or propionate. The glyoxylate shunt genes have not only been annotated, but shown to function as their heterologous expression complemented a glyoxylate deficient *C. glutamicum* mutant and their overexpression improved (*R*)-acetoin production by a metabolically engineered strain of *B. methanolicus* (Drejer et al., 2020). The genome of *B. methanolicus* also encodes propionyl-CoA carboxylase and methylmalonyl-CoA mutase, thus, providing ability to grow on propionic acid. The two chromosomally encoded and the plasmid encoded NAD^+^ dependent methanol dehydrogenases have been shown to oxidize other alcohols (ethanol, propanol, butanol, isopropanol, 1,2-propanediol, and 1,3-propanediol) besides methanol (Krog et al., 2013). This may increase the carbon source spectrum to include ethanol besides acetate, propanol, propionate, and possibly more alcohols that require gluconeogenesis to support growth of *B. methanolicus*. Future work has to address if the amino acid changes introduced here into FBA^P^ affect the ability of *B. methanolicus* to grow with these C2 and C3 carbon sources.

In *B. methanolicus*, both aldolase enzymes FBA^C^ and FBA^P^ do not only cleave FBP (glycolysis) and synthesize it from GAP and DHAP (gluconeogenesis), but they have a third role in the so-called SBPase variant of the RuMP cycle that was shown to operate *in vivo* by ^13^C-labeling experiments (Delépine et al., 2020). FBA^P^ and FBA^C^ are able to synthesize seduheptolose-1,7-bisphosphate from DHAP and erythrose 4-phosphate, which was revealed by a coupled discontinuous enzyme assay and subsequent LC-MS/MS analysis (Stolzenberger et al., 2013b). Only the plasmid-encoded phosphatase enzyme GlpX^P^ is active as FBPase and SBPase, whereas GlpX^C^ was only found to be active as FBPase (Stolzenberger et al., 2013b). It remains to be studied if the amino acid changes introduced here into FBA^P^ and FBA^C^ affect their activities in the synthesis of seduheptolose-1,7-bisphosphate from DHAP and erythrose 4-phosphate and the consequences for their roles in the SBPase variant of the RuMP cycle.

In several pathogenic bacteria, genetic studies have shown that loss of the *fba* gene led to a loss of viability of the organism (Gerdes et al., 2003; Kobayashi et al., 2003; Baba et al., 2006; Capodagli et al., 2014). Beyond that, it was recently reported that FBA plays a direct role in the transcriptional regulation of the *katG* and *rpoA* genes, which code for a catalase and an RNAP subunit, respectively, in *Francisella tularensis* (Ziveri et al., 2017). Therefore, FBA is suspected to be involved in the control of host redox homeostasis and inflammatory immune response (Ziveri et al., 2017). Furthermore, in the yeast *S. cerevisiae* the class II FBA, in addition to its function in glycolysis, physically interacts with RNAP III and plays a role in controlling its transcription (Cieśla et al., 2014; Ziveri et al., 2017). It can therefore be assumed that the FBAs in *B. methanolicus* will also take on further regulatory tasks that have not been discovered yet. This underlines the importance to study the function of FBAs more deeply, especially in poorly characterized methylotrophs to better understand and, in turn, engineer the metabolism. The present study gives first insights on the activity alternation from FBA^C^ toward FBA^P^ and vice versa by the kinetic parameters shown for the double mutant FBA^C; E51V, T140R^ and FBA^P; V51E, R140T^ both in FBP cleavage and synthesis direction. The expectation of the double mutant FBA^P; V51E, R140T^ leading to a change in reactivity of FBA^P^ toward a more glycolytic role could not be confirmed due to lack of activity detected in said mutant in glycolysis. However, an improved gluconeogenic FBA was engineered with FBA^C; E51V, T140R^, overcoming the major gluconeogenic FBA^P^.

CRISPR interference mediated gene repression of *fba*^C^ and *fba*^P^ revealed compensatory expression of the paralogous gene that was not targeted by CRISPRi (Figure 3). Since an increase in RNA levels of the non-targeted *fba* could be observed, the mechanism may either involve transcriptional regulation or mRNA degradation control. Up to date, transcriptional regulatory proteins affecting the expression of *fba*^C^ or the *fba*^P^*-tkt*^P^ operon have not been described. Similarly, *cis*-regulatory RNA elements such as riboswitches have not been identified in their 5'UTRs (Irla et al., 2015). However, methylotrophic genes present in the natural pBM19 plasmid including the *fba*^P^*-tkt*^P^ operon, but notably not *fba*^C^, were reported to show increased RNA levels in the presence of methanol (Jakobsen et al., 2006; Heggeset et al., 2012) and it is plausible that the underlying regulatory mechanism may be relevant to explain the compensatory induction of the *fba*^P^-*tkt*^P^ operon upon CRISPRi mediated repression of *fba*^C^. The compensatory induction of *fba*^C^ when *fba*^P^ is targeted by CRISPRi is even less clear. The observed phenomena in response to genetic perturbation by CRISPRi may be due to physiological robustness that has been described as the persistence of certain characteristics or traits in a biological system under perturbations or conditions of uncertainty (Kitano, 2004; Stelling et al., 2004; Félix and Wagner, 2008). The observed changes in RNA levels of *fba*^C^ and *fba*^P^ indicate robustness by genetic compensation (Lynch and Conery, 2000; Gu et al., 2003; Plata and Vitkup, 2014; Bunton-Stasyshyn et al., 2019) rather than by alternative signaling or metabolic pathways (Wagner, 2000; Papp et al., 2004; Plata and Vitkup, 2014). Genetic robustness typically involves compensatory genes that are paralogs (Lynch and Conery, 2000; Bunton-Stasyshyn et al., 2019), as is the case of *fba*^C^ and *fba*^P^. The CRISPRi approach used here allowed for gene repression of *fba*^C^ and *fba*^P^. However, in the absence of CRISPR genome editing or other methods for gene deletion in *B. methanolicus*, it remains unclear if *fba*^C^ and/or *fba*^P^ are essential or conditionally essential for methylotrophic growth, and if there are additional aldolase enzymes that may compensate for their absence (*μ* = 0.36 h^−1^, 0.35 h^−1^; Figure 2). Genetic robustness appears not to involve all methylotrophic genes since CRISPRi repression of *tkt*^P^ did neither lead to increased *tkt*^C^ RNA levels nor did CRISPRi targeting of *tkt*^C^ increase *tkt*^P^ RNA levels (Figure 4). As the transketolases TKT^P^ and TKT^C^ share very similar kinetic parameters (Markert et al., 2014) they would be well suited for genetic compensation. It is plausible that the limited CRISPRi mediated repression of *tkt* (about 2-fold, Figure 4) did not reduce TKT enzyme levels below a threshold that might trigger genetic compensation. Future work with the aim of improving the genetic toolbox of *B. methanolicus* will have to include making use of stronger and gratuitous inducers in the CRISPRi system and, eventually, to develop tools for genetic modifications in this organism. In this study, the currently available CRISPRi system was successfully employed to demonstrate the first loss of function analysis of a methylotrophic target gene and thus improve the characterization of key RuMP enzymes in *B. methanolicus*.

## Data Availability Statement

Crystal structures have been submitted to PDB with accession codes 7NC7 and 7NCC. The original contributions presented in the study are included in the article/Supplementary Material, further inquiries can be directed to the corresponding author.

## Author Contributions

KS, ML, and LK carried out the genetics and biochemistry experiments of the present study. OE and LZ performed structure elucidation experiments. All authors analyzed the data. VW coordinated the study. KS and ML drafted the manuscript. All authors revised the manuscript and VW finalized the manuscript. All authors contributed to the article and approved the submitted version.

### Conflict of Interest

The authors declare that the research was conducted in the absence of any commercial or financial relationships that could be construed as a potential conflict of interest.
