# Supplementary material for: Interrogating the Role of the Two Distinct Fructose-Bisphosphate Aldolases of Bacillus methanolicus by Site-Directed Mutagenesis of Key Amino Acids and Gene Repression by CRISPR Interference
Source: Front Microbiol. 2021 Apr 30;12:669220. doi: 10.3389/fmicb.2021.669220 (PMC8119897; doi:10.3389/fmicb.2021.669220)
Supplement: Supplementary file 1 [file Data_Sheet_1.pdf]

Supplementary Material

Interrogating the Role of the Two Distinct Fructose-Bisphosphate Aldolases of *Bacillus methanolicus* by Site-Directed Mutagenesis of Key Amino Acids and Gene Repression by CRISPR Interference

Kerstin Schultenkämper[1], Desirée D. Gütle[2], Marina Gil López[1†], Laura B. Keller[1], Lin Zhang[3] Oliver Einsle[3], Jean-Pierre Jacquot[2] and Volker F. Wendisch[1*]

[1] Genetics of Prokaryotes, Faculty of Biology & CeBiTec, Bielefeld University, 33615 Bielefeld, Germany

[2] Univ Lorraine, INRAE, IAM, F-54000 Nancy, France

[3] Institute for Biochemistry, Albert-Ludwigs-University Freiburg, 79104 Freiburg, Germany

*** Correspondence:**
Volker F. Wendisch
volker.wendisch@uni-bielefeld.de

[†] Present address: Marina Gil López, Department of Biotechnology and Food Science, Norwegian University of Science and Technology (NTNU), Trondheim, Norway

Supplementary tables

Table S1: Bacterial strains and plasmids used in this study.

Strain	Relevant characteristics	Reference
Escherichia coli DH5α	General cloning host, F- *thi*-1 *endA*1 *hsdR*17(r$^-$, m$^-$) *supE*44 _*lacU*169 (80*lacZ*_M15) *recA*1 *gyrA*96 *relA*1	(Hanahan, 1983)
Escherichia coli BL21 (DE3)	Protein production host, F- *ompT hsdSB* (r_B− m_B−) *gal dcm* (DE3)	(Studier and Moffatt, 1986)
Bacillus methanolicus MGA3	Wild type strain (ATCC 53907)	(Schendel et al., 1990)
**Plasmid**		
pET16b	Ap^R; T7*lac*; pBR322 origin, vector for N-terminal His-tagged protein overproduction	Novagen
pET16b-*fba*^{C}	Ap^R; T7*lac*; pBR322 origin, *fba*^{C} sequence was inserted in *Nde*I CA^TATG cloning site	(Stolzenberger et al., 2013a)
pET16b-*fba*^{P}	Ap^R; T7*lac*; pBR322 origin, *fba*^{P} sequence was inserted in *Nde*I CA^TATG cloning site	(Stolzenberger et al., 2013a)
pET16b-*glpX*^{C}	Ap^R; T7*lac*; pBR322 origin, g*lpX*^{C} sequence from *B. methanolicus* was inserted in *Nde*I CA^TATG cloning site	(Stolzenberger et al., 2013a)
pET16b-*fba*^{C}-*sdm1*	pET16b derivative for the production of *B. methanolicus* His_{10}-tagged Fba^C E51V mutant from *E. coli* BL21 (DE3), Ap^R; T7*lac*; pBR322 origin SDM of *fba*^{C} E51V	This study
pET16b-*fba*^{C}-*sdm2*	pET16b derivative for the production of *B. methanolicus* His_{10}-tagged Fba^C T140R mutant from *E. coli* BL21 (DE3), Ap^R; T7*lac*; pBR322 origin, SDM of *fba*^{C} T140R	This study
pET16b-*fba*^{C}-*sdm5*	pET16b derivative for the production of *B. methanolicus* His_{10}-tagged Fba^C E51V/T140R mutant from *E. coli* BL21 (DE3), Ap^R; T7*lac*; pBR322 origin, SDM of *fba*^{C} E51V/T140R	This study
pET16b-*fba*^{P}-*sdm3*	pET16b derivative for the production of *B. methanolicus* His_{10}-tagged Fba^P V51E mutant from *E. coli* BL21 (DE3), Ap^R; T7*lac*; pBR322 origin, SDM of *fba*^{P} V51E	This study
pET16b-*fba*^{P}-*sdm4*	pET16b derivative for the production of *B. methanolicus* His_{10}-tagged Fba^P R140T mutant from *E. coli* BL21 (DE3), Ap^R; T7*lac*; pBR322 origin, SDM of *fba*^{P} R140T	This study
pET16b-*fba*^{P}-*sdm6*	pET16b derivative for the production of *B. methanolicus* His_{10}-tagged Fba^P V51E/R140T mutant from *E. coli* BL21 (DE3), Ap^R; T7*lac*; pBR322 origin, SDM of *fba*^{P} V51E/R140T	This study
pET28a-*glpX*	pET28a derivative for the production of *C. glutamicum* His-tagged GlpX from *E. coli* BL21 (DE3), Knt^R; T7*lac*; pBR322 origin, *glpX* sequence from *C. glutamicum* was inserted in *Nde*I CA^TATG cloning site	(Rittmann et al., 2003)

pNW33Nkan	Cm^R, Km^R; pNW33N derivative in which the kanamycin-resistance gene was inserted	(Irla et al., 2016)
piCas	Cm^R, Km^R; pNW33N derivative, m2p controlled expression of *dcas9*, followed by terminator sequence of *S. pyogenes*. Contains another m2p promoter, which lacks 5'UTR, followed by *Ava*I/*Xba*I cloning site and dCas9 handle and terminator sequence of *S. pyogenes*	(Schultenkämper et al., 2019)
piCas-*tfba*^{C}	Cm^R, Km^R; piCas derivative, 20 bp complementary to *fba*^{C} gene was inserted in *Ava*I/*Xba*I cloning site.	This study
piCas-*tfba*^{P}	Cm^R, Km^R; piCas derivative, 20 bp complementary to *fba*^{P} gene was inserted in *Ava*I/*Xba*I cloning site.	This study
piCas-*ttkt*^{C}	Cm^R, Km^R; piCas derivative, 20 bp complementary to *tkt*^{C} gene was inserted in *Ava*I/*Xba*I cloning site.	This study
piCas-*ttkt*^{P}	Cm^R, Km^R; piCas derivative, 20 bp complementary to *tkt*^{P} gene was inserted in *Ava*I/*Xba*I cloning site.	This study

Cm^R, chloramphenicol resistance; Km^R, kanamycin resistance; Ap^R, ampicillin resistance;

Table S2: Oligonucleotide sequences used in the present study.

Oligonucleotide ID	Characteristic	Sequence [5'-3']
tfbaC_fwd	Fwd primer for annealing oligonucleotides and synthesizing 20 bp region complementary to *fba*^{C} as target for CRISPRi with overlaps to piCas plasmid	*aaacgttttatgataaatat*aatatctgcccccagctctt
tfbaC_rev	Rev primer for annealing oligonucleotides and synthesizing 20 bp region complementary to *fba*^{C} as target for CRISPRi with overlaps to piCas plasmid	*tttctagctctaaaactcga*aagagctgggggcagatatt
tfbaP_fwd	Fwd primer for annealing oligonucleotides and synthesizing 20 bp region complementary to *fba*^{P} as target for CRISPRi with overlaps to piCas plasmid	*aaacgttttatgataaatat*ccatggacagaacctaacgc
tfbaP_rev	Rev primer for annealing oligonucleotides and synthesizing 20 bp region complementary to *fba*^{P} as target for CRISPRi with overlaps to piCas plasmid	*tttctagctctaaaactcga*gcgttaggttctgtccatgg
ttktC_fwd	Fwd primer for annealing oligonucleotides and synthesizing 20 bp region complementary to *tkt*^{C} as target for CRISPRi with overlaps to piCas plasmid	*aaacgttttatgataaatat*gatggaaagtgtacgtatcg
ttktC_rev	Rev primer for annealing oligonucleotides and synthesizing 20 bp region complementary to *tkt*^{C} as target for CRISPRi with overlaps to piCas plasmid	*tttctagctctaaaactcga*cgatacgtacactttccatc
ttktP_fwd	Fwd primer for annealing oligonucleotides and synthesizing 20 bp region complementary to *tkt*^{P} as target for CRISPRi with overlaps to piCas plasmid	*aaacgttttatgataaatat*gatcaatatctattttttgt
ttktP_rev	Rev primer for annealing oligonucleotides and synthesizing 20 bp region complementary to *tkt*^{P} as target for CRISPRi with overlaps to piCas plasmid	*aaacgttttatgataaatat*gatcaatatctattttttgt
proI_fwd	Fwd primer for amplifying *proI of Bacillus methanolicus*, quality check for contaminating genomic DNA in RNA samples	caaggccgcttgaaaaggggga aatgacaaatgaagaagcttactt ttgtc
proI_rvs	Rev primer for amplifying *proI of Bacillus methanolicus*, quality check for contaminating genomic DNA in RNA samples	gcggccgcggggtacccgggg atccttactgctttactgttactg
parA_fwd	qRT-PCR fwd primer for analysis for *parA* expression	tccagcctgaaggatatagc
parA_rev	qRT-PCR rev primer for analysis for *parA* expression	tcttcggcactgttgaagga
fbaC_fwd	qRT-PCR fwd primer for analysis for *fba*^{C} expression	agccgttcagtctgttttc

fbaC_rev	qRT-PCR rev primer for analysis for *fba*^{C} expression	catcccccatttatcacattcc
fbaP_fwd	qRT-PCR fwd primer for analysis for *fba*^{P} expression	cggtggacaagaagatgatgtag
fbaP_rev	qRT-PCR rev primer for analysis for *fba*^{P} expression	acgcaggtgcaaagcag
dCas9_fwd	qRT-PCR fwd primer for analysis for *dcas9* expression	cgtcgccgttatactggttg
dCas9_rev	qRT-PCR rev primer for analysis for *dcas9* expression	ctatcgccttgtccagacac
tktC_fwd	qRT-PCR fwd primer for analysis for *tkt*^{C} expression	gagaagacggaccaacacac
tktC_rev	qRT-PCR rev primer for analysis for *tkt*^{C} expression	taggagcaacgcatcaggag
tktP_fwd	qRT-PCR fwd primer for analysis for *tkt*^{P} expression	aaagaagcagcagagaagaag
tktP_rev	qRT-PCR rev primer for analysis for *tkt*^{P} expression	cgacacggtaaacaggaac
SDM1_fwd	Backbone amplification of pET16b-*fba*^{C} with a mutation in position 51 from E to V (SDM1), fwd primer	tcattttaggagtttctgtgggcgccggccgctatatgg
SDM1_rev	Backbone amplification of pET16b-*fba*^{C} with a mutation in position 51 from E to V (SDM1), rev primer	catatagcggccggcgcccacagaaactcctaaaatgac
SDM2_fwd	Backbone amplification of pET16b-*fba*^{C} with a mutation in position 140 from T to R (SDM2), fwd primer	aagcagagcttggacgcgttggcggccaagag
SDM2_rev	Backbone amplification of pET16b-*fba*^{C} with a mutation in position 140 from T to R (SDM2), rev primer	tcttggccgccaacgcgtccaagctctgcttc
SDM3_fwd	Backbone amplification of pET16b-*fba*^{P} with a mutation in position 51 from V to E (SDM 3), fwd primer	gttattatcggggtatctgaaggtgctgctaattacatg
SDM3_rev	Backbone amplification of pET16b-*fba*^{P} with a mutation in position 51 from V to E (SDM 3), rev primer	catgtaattagcagcaccttcagataccccgataataac
SDM4_fwd	Backbone amplification of pET16b-*fba*^{P} with a mutation in position 140 from R to T (SDM4), fwd primer	ggcagagctaggtaccatcggtggacaag
SDM4 rev	Backbone amplification of pET16b-*fba*^{P} with a mutation in position 140 from R to T (SDM4), rev primer	cttgtccaccgatggtacctagctctgcc
P212	Sequencing primer of pET16a plasmids (fwd)	gctaacgcagtcaggcaccgtgta
P213	Sequencing primer of pET16a plasmids (rev)	gactcactataggggaattgtgagcg

Overlapping regions are shown in italics; fwd: forward; rev: reverse; SDM: site directed mutagenesis.

Table S3: SDMs characteristics.

**Name**	**Performed mutation(s)**	**Primer pair**	**Template**	**Relevant characteristics**
**FBA$^{C; E51V}$**	FBA^{C} E51V	SDM1_fwd/ SDM2_rev	pET16b-*fba*^{C}	Mutation in FBP binding site
**FBA$^{C; T140R}$**	FBA^{C} T140R	SDM2_fwd/ SDM2_rev	pET16b-*fba*^{C}	Mutation in Zinc binding site
**FBA$^{P; V51E}$**	FBA^{P} V51E	SDM3_fwd/ SDM3_rev	pET16b-*fba*^{P}	Mutation in FBP binding site

**FBA$^{P; R140T}$**	FBA^{P} R140T	SDM4_fwd/<br>SDM4_rev	pET16b-*fba*^{P}	Mutation in Zinc binding site
**FBA$^{C; E51V,T40R}$**	FBA^{C}<br>E51V/T140R	SDM2_fwd/<br>SDM2_rev	pET16b-*fba*$^{C-}$<br>*sdm1*	Mutation in FBP and Zinc binding site (Double mutant)
**FBA$^{P; V51E, R140T}$**	FBA^{P}<br>V51E/R140T	SDM3_fwd/<br>SDM3_rev	pET16b-*fba*$^{P-}$<br>*sdm4*	Mutation in FBP and Zinc binding site (Double mutant)

Table S4: Amino acid sequences of FBA$^{C/P}$ and SDMs.

Name	Sequence
**FBA^{C}**	MPLVSMTEMLNKAKAEGYAVGQFNLNNLEFTQAILLAAEEEKSPVILGVS**E**GAGRY<br>MGGFKTVVNMVKGLMEDYKITVPVAIHLDHGSSFEKCKEVIDAGFTSVMIDASHHPF<br>EENVEVTKKVVEYAHARGVSVEAELG**T**VGGQEDDVIADGVIYADPKECEELVKRTGI<br>DCLAPALGSVHGPYKGEPNLGFKEMEEIGRITGVPLVLHGGTGIPTKDIQRAISLGTAK<br>INVNTENQIASAKKVREVLAENPNMYDPRKYLGPARDAIKETVIGKMREFGSSGKA
**FBA$^{C; E51V}$**	MPLVSMTEMLNKAKAEGYAVGQFNLNNLEFTQAILLAAEEEKSPVILGVS**V**GAGRY<br>MGGFKTVVNMVKGLMEDYKITVPVAIHLDHGSSFEKCKEVIDAGFTSVMIDASHHPF<br>EENVEVTKKVVEYAHARGVSVEAELG**T**VGGQEDDVIADGVIYADPKECEELVKRTGI<br>DCLAPALGSVHGPYKGEPNLGFKEMEEIGRITGVPLVLHGGTGIPTKDIQRAISLGTAK<br>INVNTENQIASAKKVREVLAENPNMYDPRKYLGPARDAIKETVIGKMREFGSSGKA
**FBA$^{C; T140R}$**	MPLVSMTEMLNKAKAEGYAVGQFNLNNLEFTQAILLAAEEEKSPVILGVS**E**GAGRY<br>MGGFKTVVNMVKGLMEDYKITVPVAIHLDHGSSFEKCKEVIDAGFTSVMIDASHHPF<br>EENVEVTKKVVEYAHARGVSVEAELG**R**VGGQEDDVIADGVIYADPKECEELVKRTG<br>IDCLAPALGSVHGPYKGEPNLGFKEMEEIGRITGVPLVLHGGTGIPTKDIQRAISLGTA<br>KINVNTENQIASAKKVREVLAENPNMYDPRKYLGPARDAIKETVIGKMREFGSSGKA
**FBA$^{C;}$ E51V,T140R**	MPLVSMTEMLNKAKAEGYAVGQFNLNNLEFTQAILLAAEEEKSPVILGVS**V**GAGRY<br>MGGFKTVVNMVKGLMEDYKITVPVAIHLDHGSSFEKCKEVIDAGFTSVMIDASHHPF<br>EENVEVTKKVVEYAHARGVSVEAELG**R**VGGQEDDVIADGVIYADPKECEELVKRTG<br>IDCLAPALGSVHGPYKGEPNLGFKEMEEIGRITGVPLVLHGGTGIPTKDIQRAISLGTA<br>KINVNTENQIASAKKVREVLAENPNMYDPRKYLGPARDAIKETVIGKMREFGSSGKA
**FBA^{P}**	MPLVSMKDMLNHGKENGYAVGQFNINNLEFGQAILQAAEEEKSPVIIGVS**V**GAANY<br>MGGFKLIVDMVKSSMDSYNVTVPVAIHLDHGPSLEKCVQAIHAGFTSVMIDGSHLPL<br>EENIELTKRVVEIAHSVGVSVEAELG**R**IGGQEDDVVAESFYAIPSECEQLVRETGVDCF<br>APALGSVHGPYKGEPKLGFDRMEEIMKLTGVPLVLHGGTGIPTKDIQKAISLGTAKIN<br>VNTESQIAATKAVREVLNNDAKLFDPRKFLAPAREAIKETIKGKMREFGSSGKA
**FBA$^{P; V51E}$**	MPLVSMKDMLNHGKENGYAVGQFNINNLEFGQAILQAAEEEKSPVIIGVS**E**GAANY<br>MGGFKLIVDMVKSSMDSYNVTVPVAIHLDHGPSLEKCVQAIHAGFTSVMIDGSHLPL<br>EENIELTKRVVEIAHSVGVSVEAELG**R**IGGQEDDVVAESFYAIPSECEQLVRETGVDCF<br>APALGSVHGPYKGEPKLGFDRMEEIMKLTGVPLVLHGGTGIPTKDIQKAISLGTAKIN<br>VNTESQIAATKAVREVLNNDAKLFDPRKFLAPAREAIKETIKGKMREFGSSGKA
**FBA$^{P; R140T}$**	MPLVSMKDMLNHGKENGYAVGQFNINNLEFGQAILQAAEEEKSPVIIGVS**V**GAANY<br>MGGFKLIVDMVKSSMDSYNVTVPVAIHLDHGPSLEKCVQAIHAGFTSVMIDGSHLPL<br>EENIELTKRVVEIAHSVGVSVEAELG**T**IGGQEDDVVAESFYAIPSECEQLVRETGVDCF<br>APALGSVHGPYKGEPKLGFDRMEEIMKLTGVPLVLHGGTGIPTKDIQKAISLGTAKIN<br>VNTESQIAATKAVREVLNNDAKLFDPRKFLAPAREAIKETIKGKMREFGSSGKA
**FBA$^{P; V51E,}$ R140T**	MPLVSMKDMLNHGKENGYAVGQFNINNLEFGQAILQAAEEEKSPVIIGVS**E**GAANY<br>MGGFKLIVDMVKSSMDSYNVTVPVAIHLDHGPSLEKCVQAIHAGFTSVMIDGSHLPL<br>EENIELTKRVVEIAHSVGVSVEAELG**T**IGGQEDDVVAESFYAIPSECEQLVRETGVDCF<br>APALGSVHGPYKGEPKLGFDRMEEIMKLTGVPLVLHGGTGIPTKDIQKAISLGTAKIN<br>VNTESQIAATKAVREVLNNDAKLFDPRKFLAPAREAIKETIKGKMREFGSSGKA

Amino acid positions 51 and 140 are highlighted in red.

Table S5: X-ray data collection and refinement statistics.

Data sets		FBA^{C}	FBA^{P}
PDB accession code		7NC7	7NCC
Space group		C 2 2 2_1	C 2 2 2_1
Cell constants	a, b, c [Å]<br>α, β, γ [°]	71.97, 98.26, 139.37<br>90, 90, 90	91.58, 122.12, 160.96<br>90, 90, 90
Wavelength [Å]		1.54	1
Resolution limits [Å]		27.41 – 2.20 (2.28 – 2.20)	45.79 – 2.00 (2.05 – 2.00)
Completeness (%)		99.8 (98.0)	99.3 (99.4)
Unique reflections		24421 (2158)	60641 (4427)
Multiplicity (%)		4.1 (4.2)	13.4 (13.7)
R_{merge}[a]		0.395 (1.006)	0.156 (0.925)
$R_{p.i.m.}$		0.229 (0.551)	0.044 (0.256)
Mean I/σ (I)		6.7 (1.7)	11.7 (3.0)
$CC_{1/2}$		0.996 (0.521)	0.999 (0.661)
**Refinement statistics**			
R_{work}[b] / R_{free}		0.192 / 0.248	0.194 / 0.258
No. atoms		4324	4614
Protein		4155	4288
Ligand/ion		20	44
Water		149	282
B-factor [Å^2]		39.13	38.72
Protein		39.12	38.26
Ligand/ion		65.05	47.25
Water		36.07	44.39
R.m.s. deviations			
bond lengths [Å]		0.014	0.018
bond angles [°]		1.66	2.13
Ramachandran plot			
Favored (%)		96.81	96.63
Allowed (%)		3.19	3.37
Outliers (%)		0	0

[a] $R_{merge} = \Sigma_{hkl} [(\Sigma_i |I_i - \langle I \rangle|) / \Sigma_i I_i]$

[b] $R_{work} = \Sigma_{hkl} ||F_{obs}| - |F_{calc}|| / \Sigma_{hkl} |F_{obs}|$

R_{free} is the cross-validation R value for a test set of 5 % of unique reflections

Supplementary figures

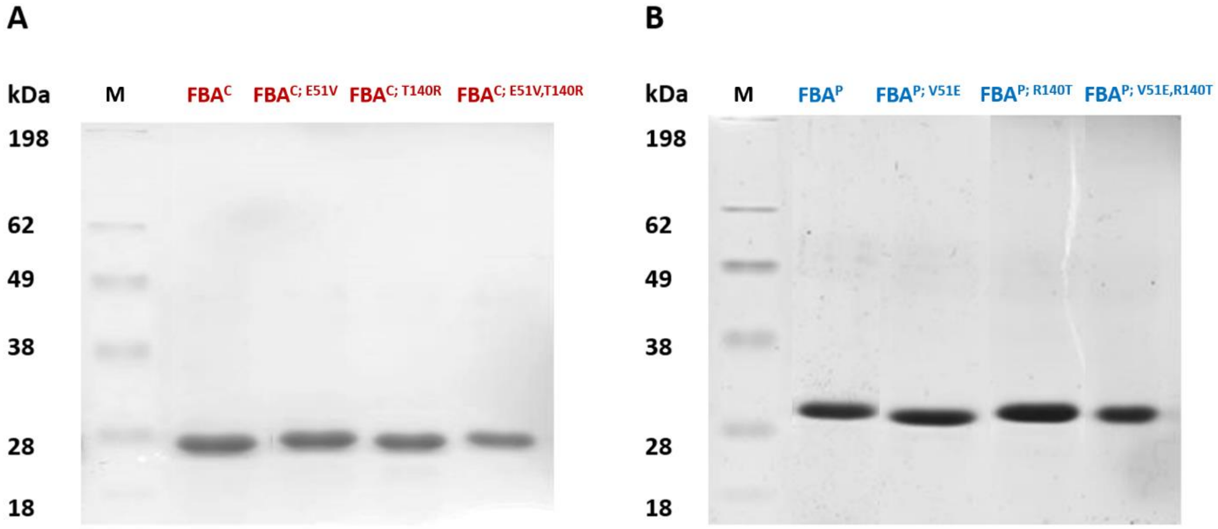

Fig. S1: Purification of distinct FBAs and mutants. SDS gels showing the purified FBA^{C} with mutants FBA$^{C; E51V}$, FBA$^{C; T140R}$ and FBA$^{C; E51V,T140R}$ (**A**), FBA^{P} with FBA$^{P; V51E}$, FBA$^{P; R140T}$ and FBA$^{P; V51E, R140T}$ (**B**) after His-Tag cleavage. Additionally, SeeBlue^{TM} pre-stained protein standard (Thermo Fisher Scientific), was applied as marker (M).

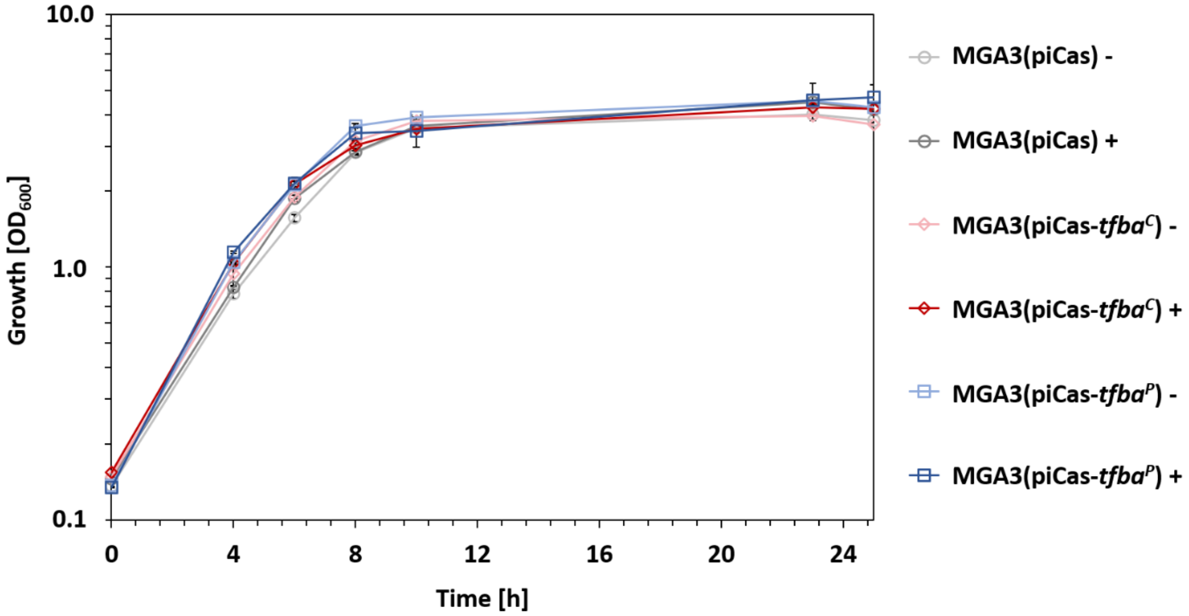

Fig. S2: Growth of *B. methanolicus* MGA3 (piCas-*tfba*$^{C/P}$) strains. Cultivation was performed with methanol as sole carbon source (-) and additionally with mannitol for *dCas9* induction (+). Error bars indicating standard deviations (n=3)

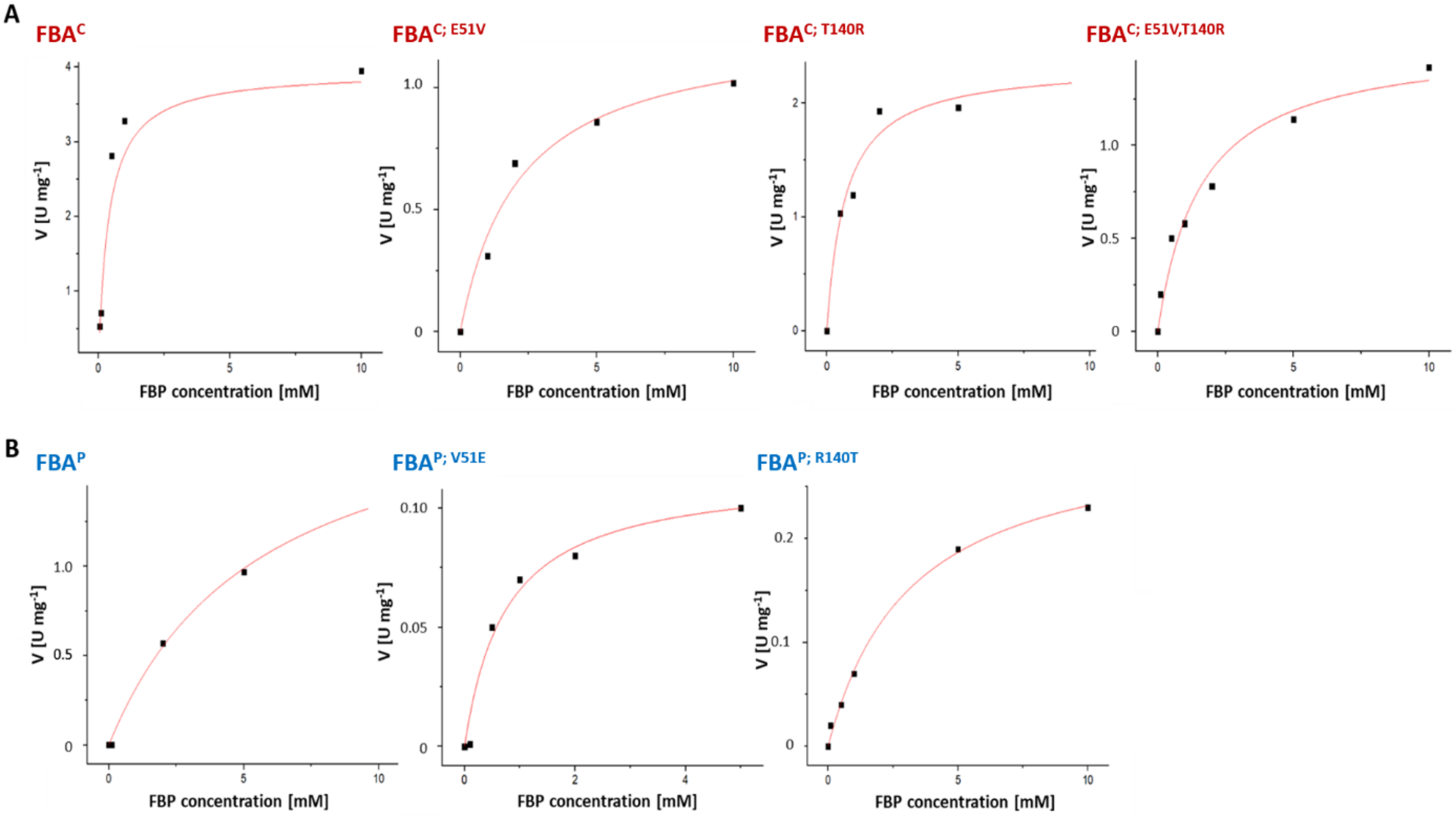

Fig. S3: Michaelis Menten Plots to determine K_M for distinct FBAs and mutants in glycolysis (FBP cleavage). The specific activity (axis of ordinate) for FBA^C and SDMs (**A**) and FBA^P and SDMs (**B**) were applied against the FBP substrate concentration (axis of abscissa). The kinetic parameters were calculated according to Michaelis Menten kinetics (Michaelis et al., 2011).

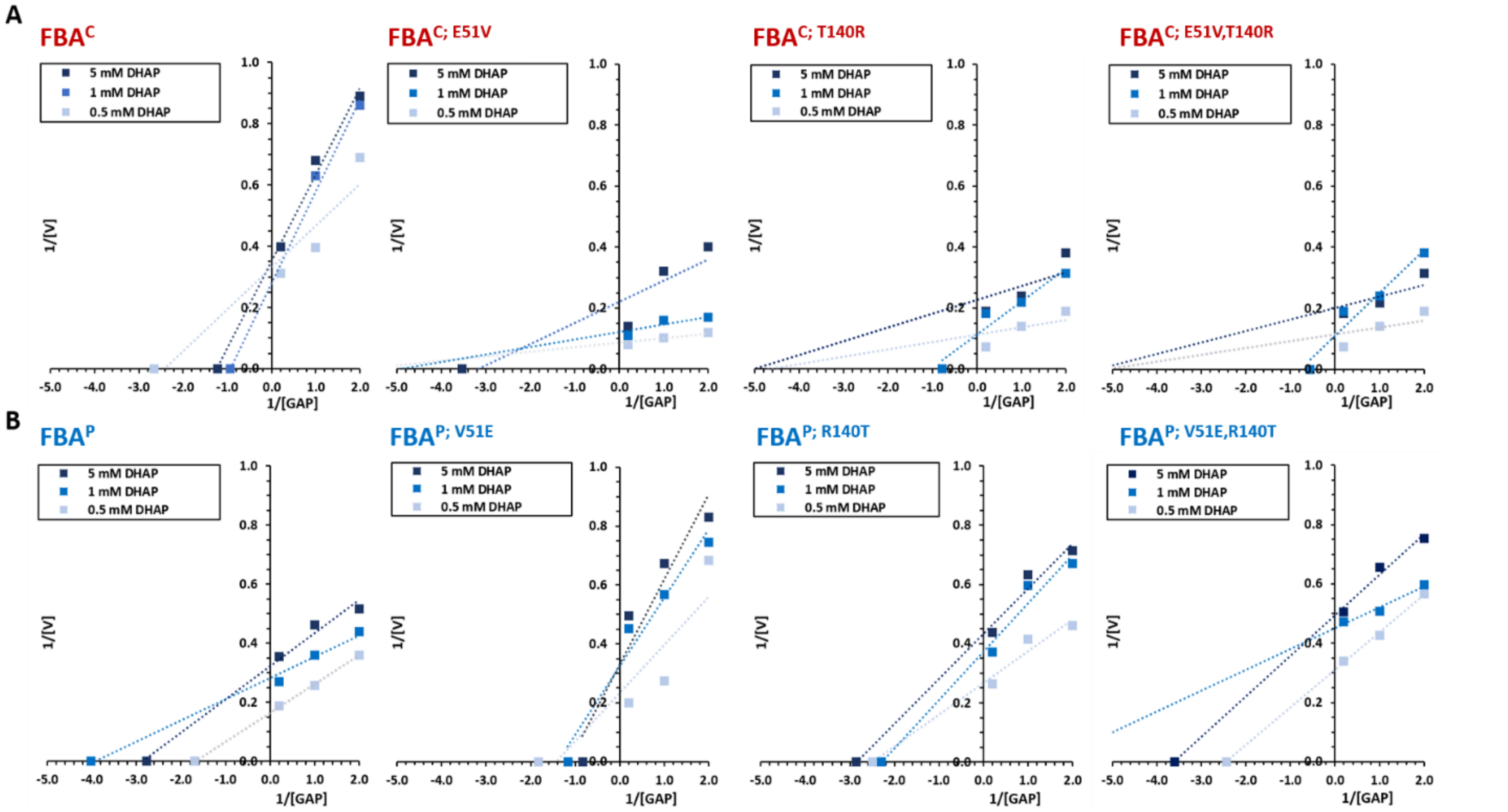

Fig. S4: Lineweaver Burk Plots to determine K_M for distinct FBAs and mutants in gluconeogenesis (FBP synthesis) with GAP as substrate. Asymmetric intersecting initial velocity patterns for an equilibrium-ordered mechanism. The reciprocals of the specific activity (axis of ordinate) for FBA^C and SDMs (**A**) and FBA^P and SDMs (**B**) were applied against the reciprocals of the GAP substrate concentration (axis of abscissa). The kinetic parameters were calculated according to Lineweaver and Burk kinetics (Lineweaver and Burk, 1934).

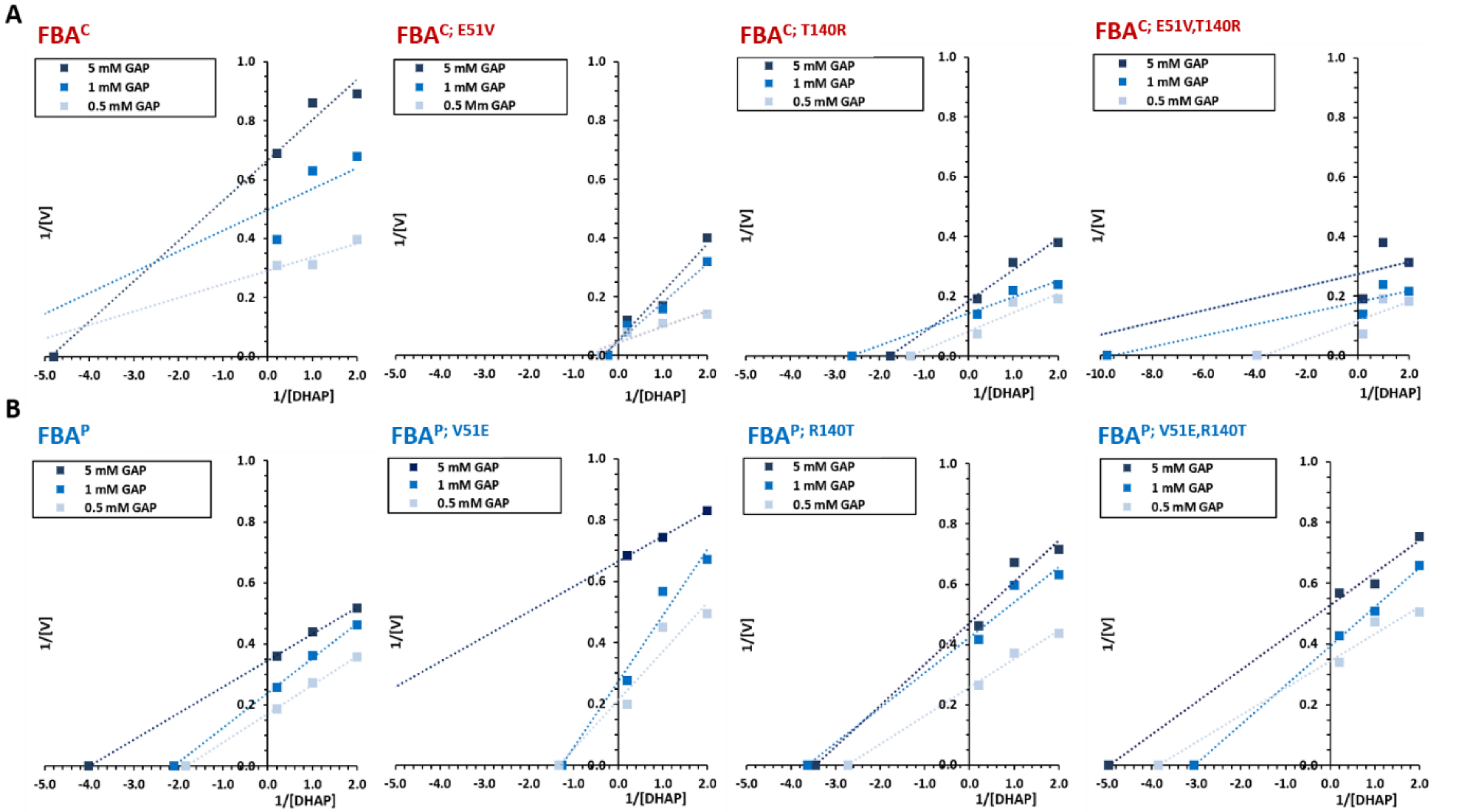

Fig. S5: Lineweaver Burk Plots to determine K_M for distinct FBAs and mutants in gluconeogenesis (FBP synthesis) with DHAP as substrate. Asymmetric intersecting initial velocity patterns for an equilibrium-ordered mechanism. The reciprocals of the specific activity (axis of ordinate) for FBA^C and SDMs (**A**) and FBA^P and SDMs (**B**) were applied against the reciprocals of the DHAP substrate concentration (axis of abscissa). The kinetic parameters were calculated according to Lineweaver and Burk kinetics (Lineweaver and Burk, 1934).
